# Microstructure and Cavitation Damage Characteristics of GX40CrNiSi25-20 Cast Stainless Steel by TIG Surface Remelting

**DOI:** 10.3390/ma16041423

**Published:** 2023-02-08

**Authors:** Ion Mitelea, Ilare Bordeaşu, Daniela Cosma (Alexa), Ion-Dragoș Uțu, Corneliu Marius Crăciunescu

**Affiliations:** 1 Department of Materials and Fabrication Engineering, Politehnica University Timisoara, Bulevardul Mihai Viteazul nr.1, 300222 Timisoara, Romania; 2 Department of Mechanical Machines, Equipment and Transports, Politehnica University Timisoara, Bulevardul Mihai Viteazul nr.1, 300222 Timisoara, Romania

**Keywords:** high Cr-Ni-Si cast stainless steel, cavitation erosion, microstructure, TIG surface remelting

## Abstract

Cavitation erosion degrades the surface of engineering components when the material is exposed to turbulent fluid flows. Under conditions of local pressure fluctuations, a nucleation of gas or vapor bubbles occurs. If the pressure suddenly drops below the vapor pressure, these bubbles collapse violently when subjected to higher pressure. This collapse is accompanied by the sudden flow of the liquid, which is manifested by stress pulses capable of causing plastic deformations on solid surfaces. Repeating these stress conditions can cause material removal and ultimately failure of the component itself. The present study aims to reduce the negative impact of this phenomenon on the mechanical systems components, using the TIG local surface remelting technique. Cavitation erosion tests were performed in accordance with the ASTM G32-2016 standard on samples taken from a cast high-alloy stainless steel. The alloy response for each melting current value was investigated by measuring mass loss as a function of cavitation attack time and by analyzing the damaged surfaces using optical and scanning electron microscopes. It was highlighted that the TIG remelted layers provide an increase in cavitation erosion resistance of 5–6 times as a consequence of the fine graining and microstructure induced by the technique applied.

## 1. Introduction

Cavitation erosion manifests itself by reducing the lifetime of numerous components that belong to mechanical systems. Degradation of parts occurs through the appearance of microcracks that develop into microcraters (pits), and finally through localized material loss [1,2,3,4].

A series of studies have been carried out to investigate the specific cavitation phenomena and the relevant physical variables [5,6,7,8,9,10]. More precisely, under certain conditions of pressure applied to a liquid, vapor bubbles can form, and following their implosion, shock waves and microjets are born that cause damage to adjacent solid surfaces [1,11,12,13,14,15].

Although the physical causes of cavitation erosion are well known, the response of the materials from which engineering components are made is a less complete field, in part because of the wide variety of possible and available materials [16,17,18].

Cavitation experiments were performed on numerous alloys, comparing and compiling their results in terms of the mass loss of samples related to the time. Correlations between these measurements and some material properties were only partially successful [1,3]. In addition, the initiation and progression of damage mechanisms are not yet fully understood. Therefore, the purpose of this study is to analyze the evolution of cavitation damage for TIG remelted surfaces of a cast, high alloy steel. This process of local surface remelting offers the advantage of simplicity, flexibility and low cost. It has many potential applications in fields such as aerospace, automobiles, nuclear reactors, ships, civil parts, etc. The material GX40CrNiSi25-20 is a cast steel, used for the manufacture of elements used for high mechanical stress and for cavitational or high temperature working environments. Among them are automotive industry: (turbochargers, manifolds, valves) and plant engineering (for oil and gas, valve bodies) [1,19].

## 2. Experimental Procedure

**Figure 1:**
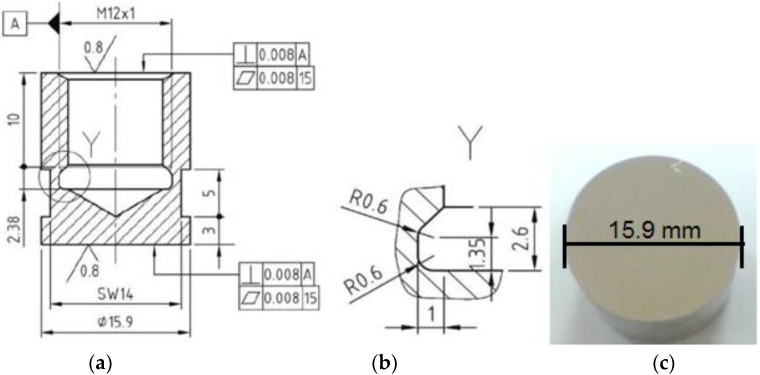
Cavitation sample: manufacturing details: (**a**) axial section through the sample, (**b**) detail of the Y area (thread clearance), (**c**) photo image of the sample surface, before the start of the cavitation attack (dimensions are in mm, according to general tolerance ISO 2768 1&2 standard).

From cast steel bars, GX40CrNiSi 25–20, (1.4848): EN 10295 (heat treated by annealing for homogenization 1100 °C/8 h/furnace), of the chemical composition C = 0.38%, Cr = 25.20%, Ni = 20.8%, Si = 1.62%, Mn = 1.49%, Mo = 0.34%, *p* = 0.031%, S = 0.027%, Fe = bal.%, cylindrical samples were made, ϕ 40 mm × 60 mm. Their surface was modified using the local TIG remelting technique, with a non-fusible electrode and using welding currents of different intensities (Is = 100 A; Is = 150 A; Is = 200 A). The other technological parameters were kept constant: electric arc voltage, Ua = 10–11 V; welding speed, vs. = 15 cm/min; electric arc length, L = 2 mm; the step between rows, *p* = 3 mm. The TIG welding equipment used for the local surface remelting of the front cylindrical samples ϕ 40 mm × 60 mm was a MW-300 inverter source from the Fronius company. Later, according to the model shown in Figure 1, cavitation samples were machined, and the expose surface was polished, on a Buehler Phoenix Beta device to a roughness Ra = 0.2–0.8 μm.

**Figure 2:**
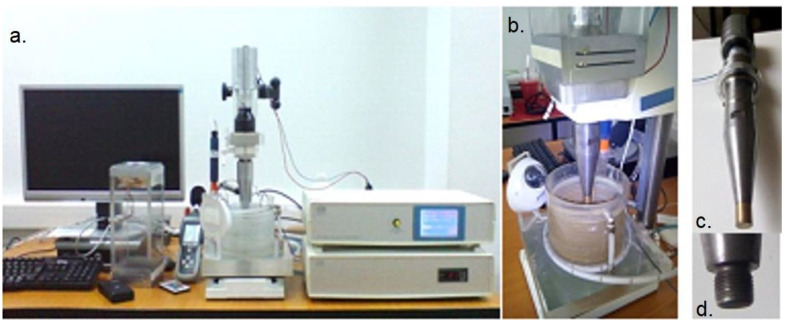
The vibrating device with piezoceramic crystals: (**a**) overview of the device with the computer control system; (**b**) image during the cavitation test (sample immersed in water); (**c**) mechanical vibration system (piezoceramic transducer with amplitude amplification system (buster, sonotrode and cavitation sample); (**d**) threaded end of the sonotrode for fixing the sample.

The experimental researches were carried out in the cavitation laboratory of the Timișoara Polytechnic University, on the vibrating device with piezoceramic crystals (Figure 2), which is controlled by a computer and equipped with an automatic control system of the functional parameters that define the cavitation hydrodynamic process. This type of device allows for the testing of different types of materials at intense cavitation erosion regimes, thus considerably reducing the samples attack duration, compared to existing situations, or using other methods of generating the cavitation phenomenon (the case of the tunnel hydrodynamic [20] or of the rotating disk device [21,22,23]).

The functional parameters of the device are:

- power of the electronic ultrasound generator, 500 W;
- vibration frequency, 20,000 ± 2% Hz;
- vibration amplitude, 50 μm;
- sample diameter: 15,9 ± 0.05 mm;
- power supply: 220 V/50 Hz;
- testing liquid: distilled water, having a temperature of 22 ± 1 °C.

The research procedure is the one described by the international standards ASTM G32-2016 [24], and the testing stages specific to the laboratory consisted of:

- weighing each sample and recording the initial mass (first on an electronic balance and then on a precision analytical balance, type Zatklady Mechaniki Precyzyjnej WP 1);
- fixing the sample in the sonotrode by threading;
- fixing the sonotrode in the vibrator support and connecting the piezoceramic transducer to the power source;
- immersing the sample in the vessel with the testing liquid (distilled or potable water from the network), to a depth of 5–10 mm;
- setting up the intermediate cavitation attack duration (5, 10 and 15 min, respectively) and starting the electronic ultrasound generator simultaneously with the cooling water recirculation system from the copper coil in the testing liquid vessel;
- after the end of each time period allocated to the cavitational attack, every sample was washed in acetone solution and dried with a blower, after which it was weighed on the electronic and precision balance to determine the eroded mass;
- after each test period, every sample was photographed using a Canon PowerShot Sx200 IS camera, 12 × Optical Zoom, whose resolution allows the damage expanding on the surface to be highlighted; they were also analyzed using an optical microscope.

According to laboratory procedure, the total duration of the cavitation attack was 165 min, divided into one period of 5 and 10 min, and 10 periods of 15 min each.

**Figure 3:**
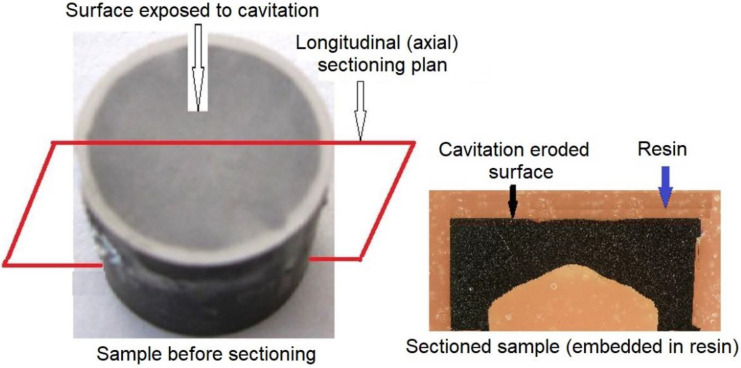
Sample preparation for optical and scanning electron microscopy investigation.

Both at intermediate attack durations and at the end of the 165 min on the degraded surfaces, the surface topography was investigated using both the optical stereomicroscope OLIMPUS SYX7 and the scanning electron microscope TESCAN VEGA 3 LMU Bruker EDX Quantax. Following the cavitation tests, the samples were longitudinally sectioned (Figure 3) and metallographically prepared for examination using both the Leica DM2700M optical microscope and the scanning electron microscope of the edge layer where erosion cracks are initiated and propagated by cavitation.

## 3. Results and Discussion

### 3.1. Cavitation Curves

**Figure 4:**
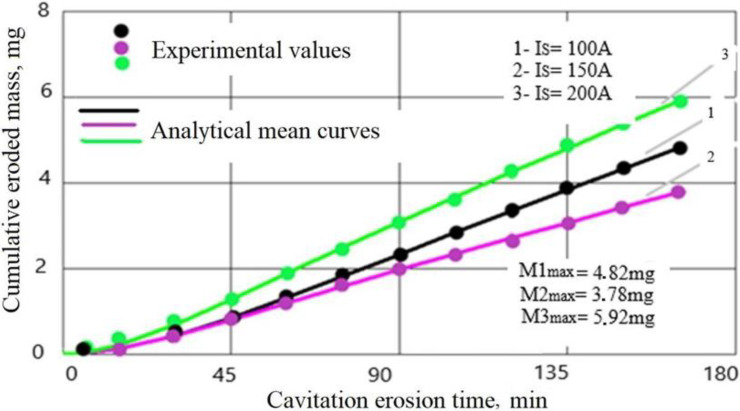
Mass losses of GX40CrNiSi25-20 steel at different remelting current intensities.

**Figure 5:**
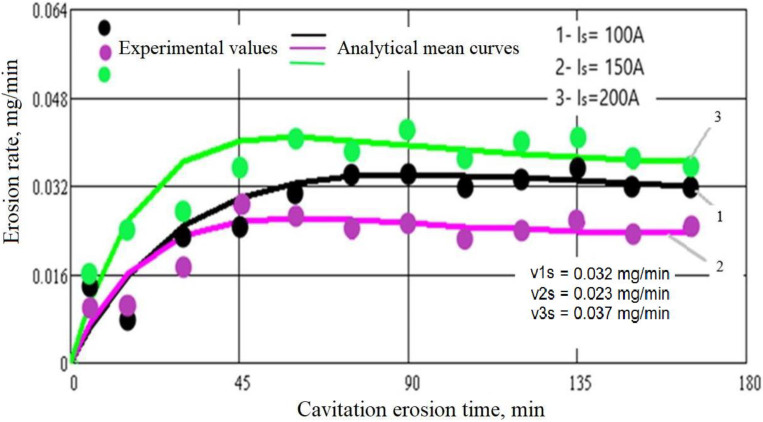
Erosion rate of GX40CrNiSi25-20 steel at different remelting current intensities.

**Figure 6:**
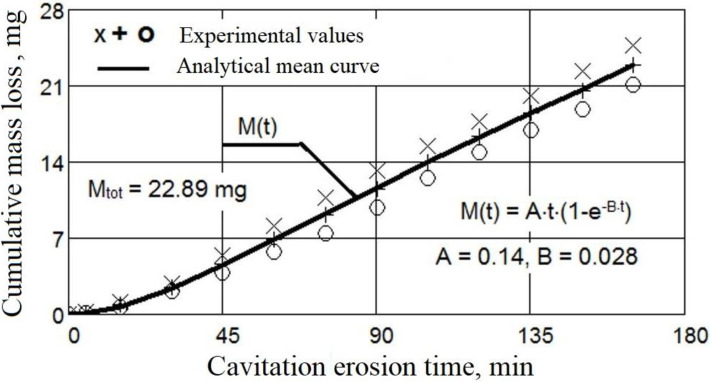
Mass losses of GX40CrNiSi25-20 steel related to the cavitation time (no remelting).

**Figure 7:**
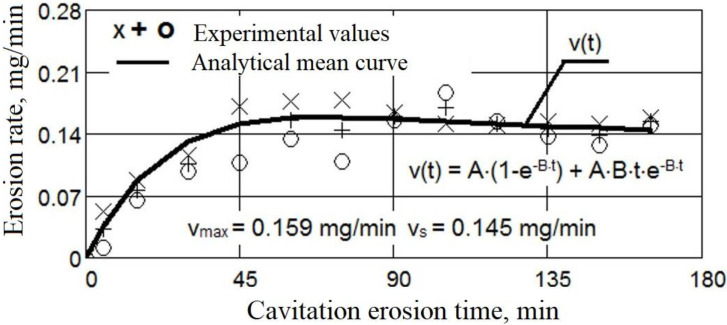
Erosion rate of GX40CrNiSi25-20 steel related to the cavitation time (no remelting).

The evolution of the mass losses and the erosion rate according to the cavitation attack time for the three values of the surface melting current are shown in Figure 4 and Figure 5. For comparison, Figure 6 and Figure 7 show the same types of curves for the samples that were not surface remelted.

The TIG process of local surface remelting achieves the modification of the microstructure without adding a new material. Following its application, it was expected to achieve homogenization and finishing of the microstructure and dissolution/redistribution of precipitates or inclusions while the properties of the substrate can be preserved. As can be observed from these diagrams, the GX40CrNiSi25-20 steel with the TIG remelted surface where an intensity current of Is = 150 A was used (samples 2 Figure 4), has the lowest mass loss compared to the same, but unmelted, steel (samples from Figure 6). For values of the remelting current of 150 A, the most favorable hardening situation occurs both through the granulation finishing and through the formation of a solid solution. The heat cycle characteristic for this remelting current value favors the precipitation of fine secondary carbides from the austenite and prevents the formation of eutectic carbides. Implicitly, the same can be said about the erosion rate, which is lower when the steel surface is remelted with a current of intensity Is = 150 A. In the case of the samples where the current of 150 A was used (samples 2 Figure 5), the mass loss is 27% lower than when using a current of 100 A (Sample 1, Figure 4) and 56% lower than when remelting with a current of 200 A (Sample 3, Figure 4). At the same time, compared to the regime with Is = 100 A, the decrease in erosion rate is over 39%, and compared to the regime with Is = 200 A, the decreasing in erosion rate is about 60%. The total cumulative mass loss for the regime with Is = 150 A (Mtot. = 3.78 mg), compared to the initial state (without remelting, Mtot. = 22.89 mg) decreases by more than six times, and the mass loss rate, according to the value of towards which the curve v(t) tends to stabilize, (vs = 0.145 mg/min. for the initial non-remelted surface, respectively vs = 0.029 mg/min. for the remelted surface at a current of 150 A), is reduced by about five times.

The approximation of the experimental values by the curves described by analytical equations, both for the mass losses (Figure 4 and Figure 6) and for the related erosion rates (Figure 5 and Figure 7), was necessary because, according to ASTM requirements G32-2016, they offer a behavior tendency and evaluation, by comparison, of the surface resistance, depending on the parameters values defined by them (Mtot.—after 165 min of cavitation attack, vmax.—the maximum of the curve v(t) and vs—final level value towards which the v(t) curve tends).

The analytical formulas were established by Pătrășcoiu a.o [20], starting from a model developed by Bordeașu et al., in 2006 [25], using a “damped”, according to which the loss curve (such as the one for loss volumes—used as an example of Patrăscoiu) is given in the equation:

M(t) = A[m_s_ − f(t)] (1)

where m_s_ represents the maximum value of mass losses, and f(t) is the solution of homogeneous linear differential equations of degree 2 with constant coefficients:

(2) $$\frac{d^{2}m}{\mathrm{dt}}+\frac{\mathrm{dm}}{\mathrm{dt}}+{\;\beta }^{2}m$$

which describes “damped” oscillation with “infinite period” and m—represents the eroded mass in time t.

Solving this last equation, considering as a variable the cumulative mass eroded during the experiment (as it is in our case), with the mathematical equation of Pătrășcoiu a.o [20,25], applying the method of least squares, the analytical form of the averinging curve of the experimental values, as follows:

M(t) = A·t·(1 − e^−B·t^) (3)

through the derivation in relation to the exposure cavitation time, the equation of mass loss rate (erosion rate) was obtained:

v(t) = A·(1 − e^−B·t^) + A·B·t·e^−B·t^ (4)

Coefficients A and B were obtained by the method of least squares.

Comparing the results shown in Figure 4 and Figure 7, it can be seen that the decrease in the cumulative masses from the end of the cavitational attack, in the case of the TIG electric arc remelting process, led to an increase in the cavitation resistance, the erosion rate and the cumulative mass losses being approximately 5–6 times higher in the unmelted samples:

$$\frac{M_{\max}}{M_{2\max}}=\frac{22.89}{3.78}=6.05$$

$$\frac{v_{s}}{v_{2s}}=\frac{0.145}{0.029}=5$$

M_2max_ și v_2s_ are the values of mass losses and rates shown in the diagrams from Figure 4 and Figure 5. In the above relations Mmax., Mtot., Mtot. and vs. are the values defined by the M(t) and v(t) curves recorded for GX40CrNiSi25-20 steel with a non-remelted surface.

### 3.2. Macro- and Micrographic Examinations

**Figure 8:**
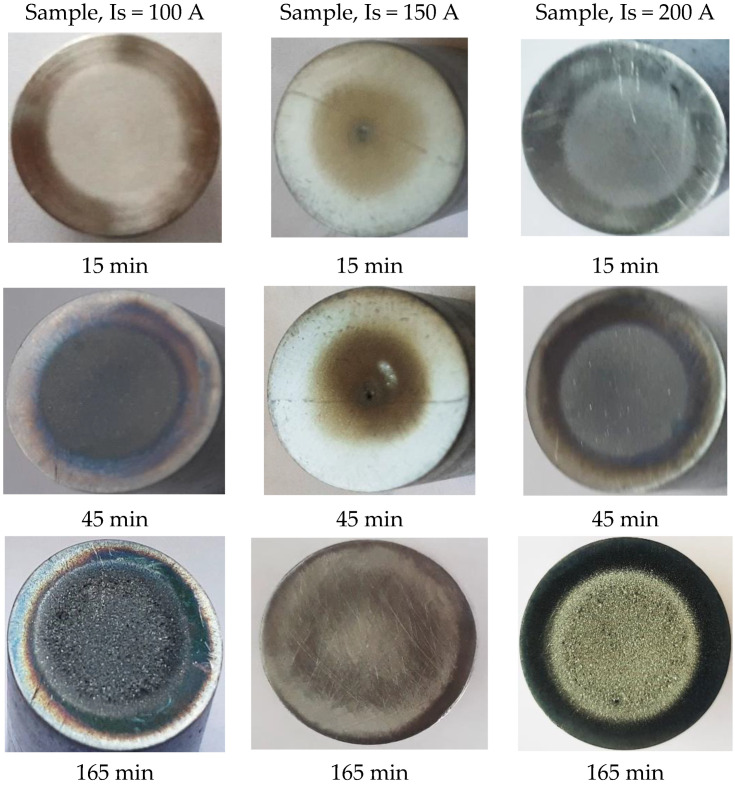
The macrographic image of the TIG surface remelted sample, with different current intensities, after the cavitation attack.

Using a Canon Power Shot SX200 IS camera, images of the samples surface were taken after each cavitation attack period to track the time evolution of erosion expanding in area and depth. Considering that the samples surfaces, from each set of three, had similar behaviors regarding degradation through cavitation erosion, in Figure 8 macroscopic images are selected, for three significant periods of time, at one of the samples, arbitrarily selected.

No images from the first two cavitation periods (of 5 and 10 min) were shown because, in this time range, the erosion mechanism consisted of the abrasive dust removal, the destruction of the roughness tip left after the finishing operation, deformations and cracks, without achieving important material expulsions.

**Figure 9:**
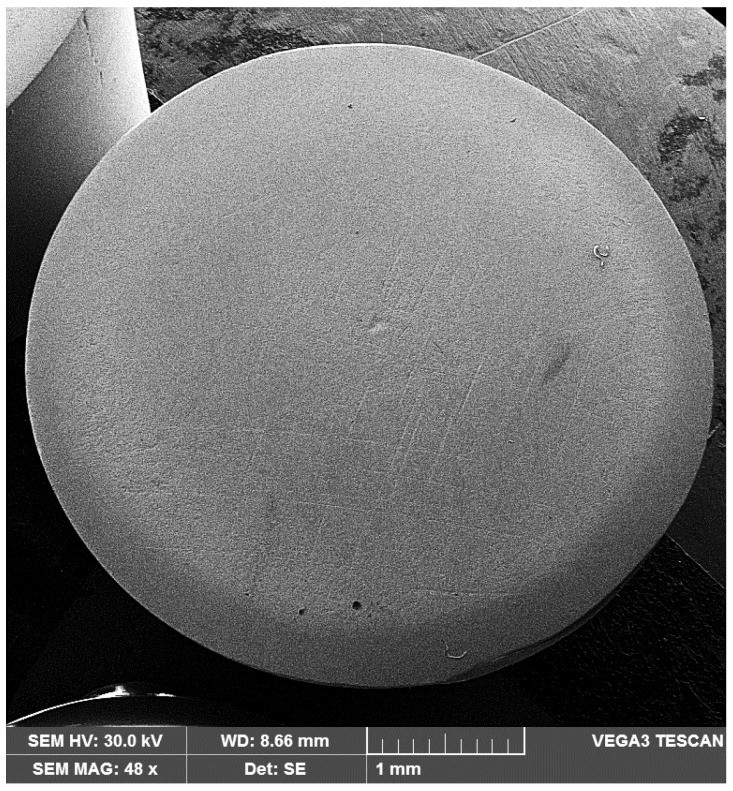
SEM macrograph of theTIG remelted surface with Is = 150 A and tested for cavitation 165 min.

The images shown in Figure 8 reveal common elements of behavior and resistance, but also differences in the cavitation surfaces destruction, as a result of the different TIG remelting parameters regime. The main observations that emerge are:

- regardless of the heat adopted regime, the erosion is manifested by the appearance of a peripheral ring, which advances towards the center surface with the increase in the duration of the cavitation attack; the damage is in the form of pitting, as a result of cyclic stresses, created by the impact with the cavitational microjets;
- as cavitation duration increases, the resulting surface roughness for the remelting current Is = 200 A is the most affected, showing that the use of this TIG regime leads to the lowest wear resistance;
- the best resistance to the vibrating cavitation demands is obtained by the remelted surface Is = 150 A, the destruction evolution in terms of area and depth being the most advantageous. The SEM image in Figure 9 proves this fact;
- the use of a remelting current Is = 100 A leads to an intermediate interaction between its other two values;
- the photographic images obtained are in full agreement with the results provided by the specific cavitation curves in Figure 4 and Figure 5.

**Figure 10:**
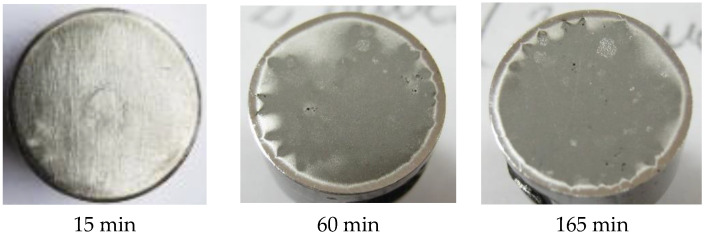
Macrographic image of the unmelted surface of GX40CrNiSi 25-20 sample.

**Figure 11:**
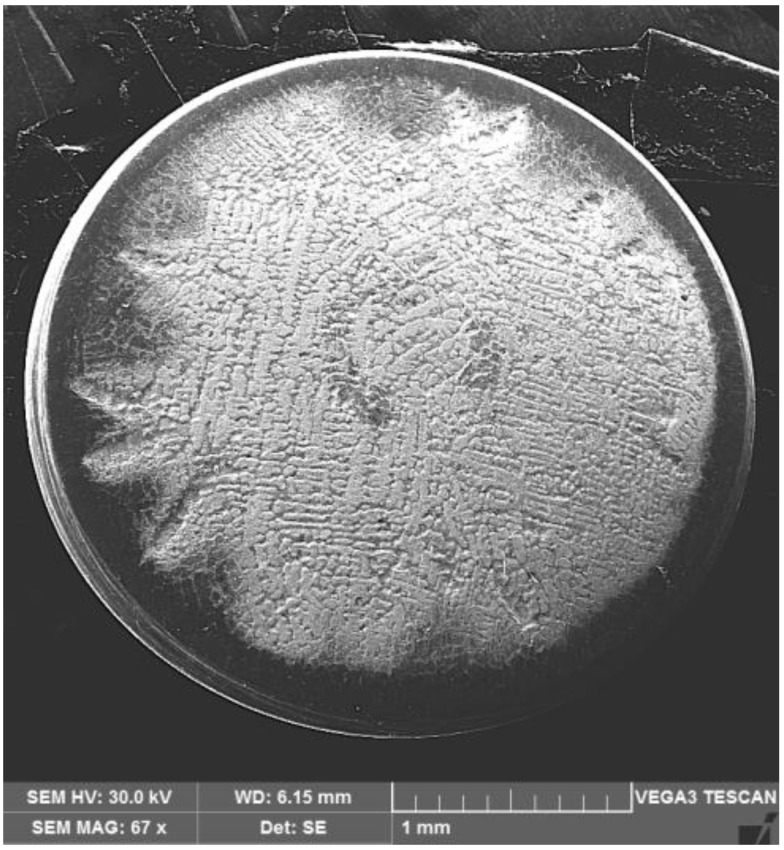
SEM macrograph of the unmelted surface tested by cavitation after 165 min.

In Figure 10, for comparison, the photo images are shown, taken at certain times, of the eroded surface for the GX40CrNiSi 25-20 steel samples, tested in the initial state, and of annealing (without remelting the surface). Figure 11 shows the SEM image of the annealed sample surfaces tested for cavitation 165 min. The jagged appearance of the outer area in the cavitated surface is due to the intradendritic microsegregations of the alloying elements present in high concentrations in the steel chemical composition. Comparing the images from Figure 9 and Figure 11 clearly demonstrates that the local TIG remelting leads to a significant improvement in the cavitation erosion resistance.

**Figure 12:**
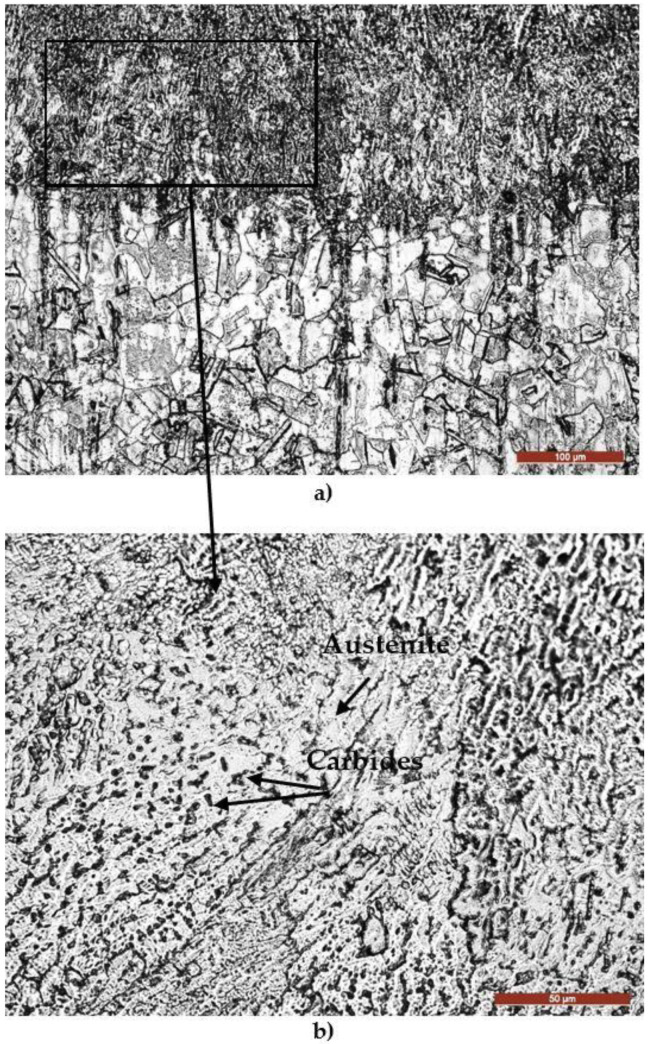
The microstructure of the layer—substrate system during the local TIG surface remelting with Is = 150 A: (**a**)—layer—substrate interface; (**b**)—remelted layer.

The micrographic examination of the longitudinal section through the cavitation samples that were TIG remelted (Figure 12) proves that this surface-ennobling process is manifested by the accentuated grain finishing and implicitly of the microstructure (austenite + carbides), with consequences for the pronounced improvement of the resistance to cavitation erosion degradation.

**Figure 13:**
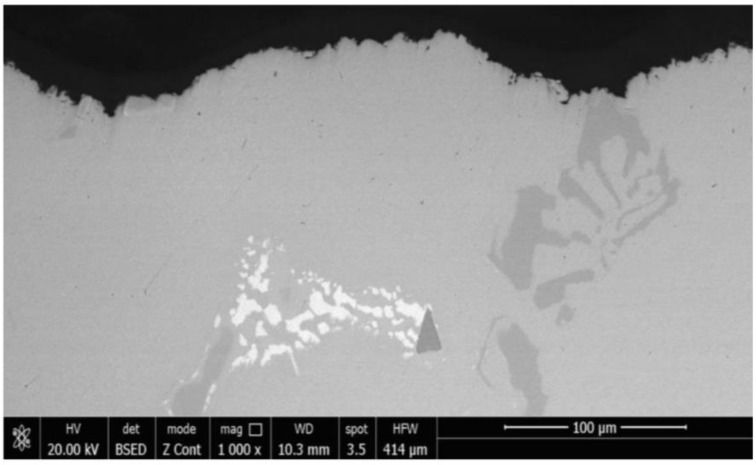
SEM microscopy of an unetched section through the surface of the reference samples (annealed).

**Figure 14:**
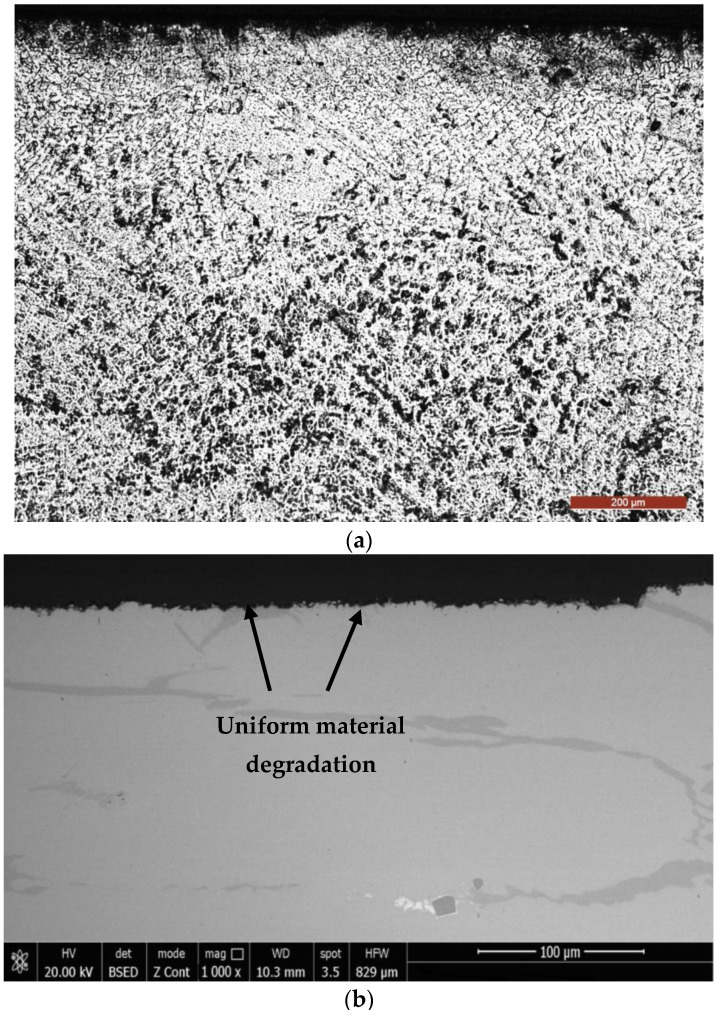
SEM microscopy through the remelted layer with Is = 150 A and after 165 min cavitation time: (**a**)—Villela etched; (**b**)—unetched.

Figure 13 and Figure 14 show some representative images that illustrate the material microstructure degradation in the annealed state, respectively, after the TIG surface remelting with a current of Is = 150 A.

They demonstrate that this form of degradation is predominantly mechanical and is characterized by a surface percussion as a result of the implosion of the gas bubbles in the liquid mass when its temperature is constant and the pressure drops to a certain critical value. Since the steel analyzed is part of a highly alloyed class, cast in pieces, it presents a heterogeneous structure (austenite + carbides) [1]

Both the carbides and the intradendritic microsegregations specific to any high-alloy steel negatively influence the mechanical properties and can constitute stress concentrators, respectively, and crack primers due to cavitational stresses.

From the presented micrographs, it can be seen that the initiation of degradation takes place on the boundaries of the austenite grains, where the carbide particles are present, microstructural constituents with the lowest resistance to cavitation. As erosion progresses, attack occurs in the solid solution matrix γ (austenite). Because of the accentuated granulation finishing and the microstructure through the local surface remelting, the appearance of the cavitation surface is much more uniform compared to the material reference state (Figure 13 and Figure 14b).

### 3.3. Topography of the Surfaces Tested for Cavitation

**Figure 15:**
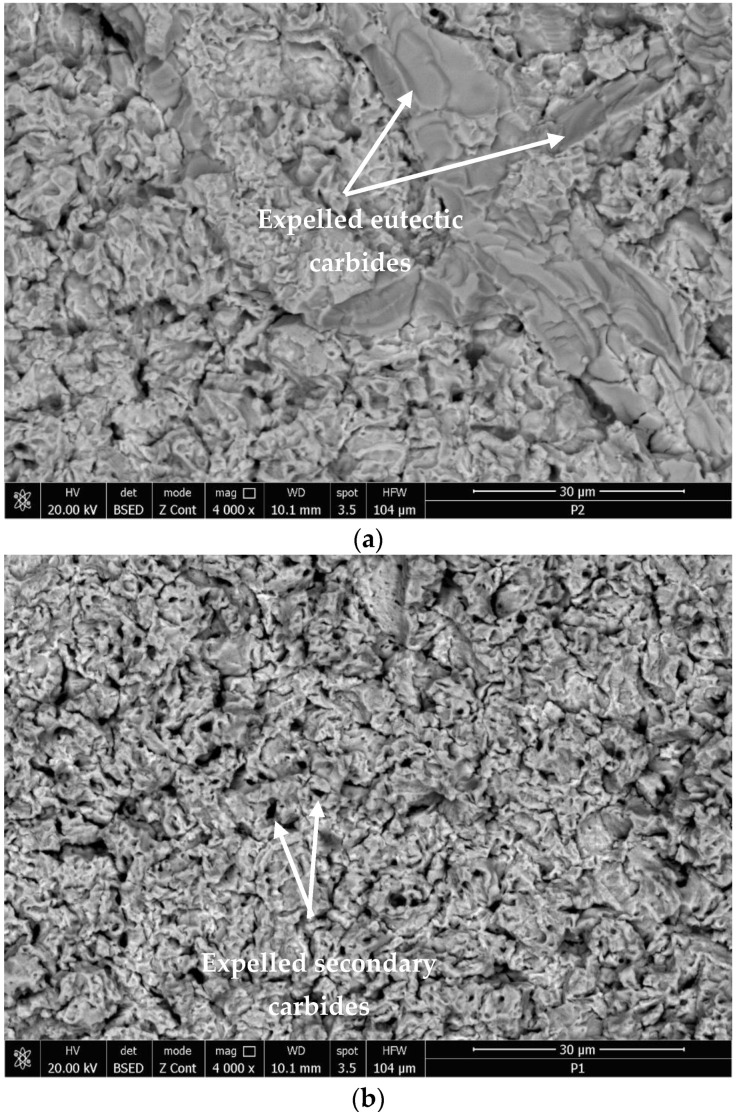
The microfractographic image of the cavition surface for 165 min. (**a**)—annealed structural state; (**b**)—TIG surface remelting with Is = 150 A.

The typical topographies of the cavitation surfaces for the samples heat-treated through annealing for homogenization and subsequently TIG surface remelted highlight a preferential degradation of the grains boundaries, where the hard and brittle carbide particles are present (Figure 15a,b). This surface modification technique leads to a structure with a high degree of fragmentation and finishing, which favors an increase in hardness and other mechanical resistance characteristics [13,14,15].

The measured hardness of the annealed surfaces, which were tested in this work, has values of 207~210 HV, and the cavitation erosion rate was 0.145 mg/min. By the remelted TIG samples with Is = 150 A, the surface hardness has values of 386~394 HV, and the erosion rate through cavitation is 0.029 mg/min. Consequently, the cavitation erosion rate of the TIG surface remelted is approx. five times higher than that of the reference material. The removal of chromium carbide particles under the action of cavitation bubbles produces high stress concentrations.

Previous research [1,25,26,27] reported that in austenitic stainless steels, the interface between chromium carbides and austenite are associated with intense stresses. Interfaces cannot undergo work hardening and consequently become brittle and fragmented through cavitation attack. At the same time, the localized strength of the austenite structural matrix increases with the degree of cold deformation during cavitation erosion. This explains the fact that following the removal of chromium carbide particles under the action of cavitational bubbles, high stress concentrations appear and the boundaries between grains are attacked more compared to solid solution grains γ.

Finishing the grain and limiting the coarse carbide precipitation after the TIG surface remelting increases the energy absorption capacity of the cavitation impact wave, which delays the cracks nucleation.

## 4. Conclusions

The surface modification of the high alloy steel GX40CrNiSi25-20 using the TIG remelting technique using currents of 100–200 A leads to a significant improvement in the cavitation erosion resistance.

The experimental results proved that for values of the remelting current of 150 A, the total cumulative mass loss (Mtot = 3.78 mg) decreases more than six times compared to the initial state (without remelting, Mtot = 22.89 mg), and the erosion rate, according to the value towards which the v(t) curve tends to stabilize, vs = 0.145 mg/min. for the initial unmelted surface and vs = 0.029 mg/min. for the remelted surface), and decreases by about five times. This hardening phenomenon obtained for remelting values of 150 mA is explained by the accentuated granulation finishing and by the favourable conditions for the formation of solid solutions

The optical and scanning electron microscope investigation of the longitudinal section through the cavitation samples that were TIG remelted proves that this surface-ennobling process causes an increase in the surface hardness from approx. 210 HV at approx. 390 HV, an accentuated granulation finishing and microstructure with consequences for the pronounced increase in the resistance to cavitation erosion degradation.

## Data Availability

Not applicable.
